# Community engagement studios to improve racial diversity and health equity in Parkinson’s

**DOI:** 10.1017/cts.2025.10119

**Published:** 2025-09-09

**Authors:** Eunyoung Kang, Dominique Woodhouse, Kandace Davis, Jane Q. Yap, Peter S. Myers, Meghan C. Campbell, Yvonne D. Hughes, Joyce E. Balls-Berry, Erin R. Foster

**Affiliations:** 1 UTHealth Houston Institute for Implementation Science, The University of Texas Health Science Center at Houston School of Public Health, Houston, TX, USA; 2 Department of Health Promotion and Behavioral Sciences, UTHealth Houston School of Public Health, Houston, TX, USA; 3 Center for Health Promotion and Prevention Research, UTHealth Houston School of Public Health, Houston, TX, USA; 4 Program in Occupational Therapy, Washington University School of Medicine, St Louis, MO, USA; 5 Department of Biomedical Ethics & Quantitative Health Sciences, Mayo Clinic, Rochester, MN, USA; 6 Office of Postdoctoral Affairs, Washington University in St Louis, St Louis, MO, USA; 7 Departments of Neurology and Radiology, Washington University School of Medicine, St Louis, MO, USA; 8 Knight Alzheimer Disease Research Center, Department of Neurology, Washington University School of Medicine, St Louis, MO, USA; 9 Program in Occupational Therapy, Department of Neurology & Department of Psychiatry, Washington University School of Medicine, St Louis, MO, USA

**Keywords:** Community engagement, Parkinson’s disease, health disparity, research methods, community engagement studios

## Abstract

**Background:**

Black or African Americans (AA) with Parkinson’s disease (PD) are underrepresented in both care and research and experience significant health disparities. The existing literature provides limited guidance on how to enhance the engagement of AA individuals in PD care and research, particularly from the perspectives of AA patients, care partners, and healthcare providers. This project aimed to (1) describe the use of Community Engagement (CE) Studios as a community-engaged research approach to inform culturally appropriate and inclusive research and (2) examine factors influencing AA engagement in PD-related activities.

**Methods:**

We conducted three CE Studios: one with AA with PD and care partners (*N* = 6), one with healthcare providers of AA with PD (*N* = 8), and one with AA with PD, care partners, and healthcare providers (*N* = 4).

**Results:**

The CE Studios informed the design (e.g., cultural appropriateness) and conduct (e.g., accessibility) of the planned PD project, as well as identifying stakeholders to engage with, improving alignment between research and the AA community. We highlighted the importance of multifaceted factors, including environmental (e.g., segregation), biological (e.g., symptoms), sociocultural (e.g., not being invited), and behavioral (e.g., empowerment) domains, which influence AA engagement.

**Conclusions:**

The CE Studios method is a feasible and useful approach for understanding the perspectives of AA in PD. It is possible to conduct an in-depth exploration of community perspectives by synthesizing comprehensive analyses and leveraging additional frameworks. These efforts include identifying barriers to engagement, recognizing locally relevant individuals, and refining PD-related care to enhance cultural appropriateness.

## Introduction

People who identify as Black or African American (AA) with Parkinson’s disease (PD) can experience significant health disparities. Compared to other racial groups, they often receive a diagnosis later, receive less standard, specialty, mental health, and rehabilitation care, and have poorer health outcomes [1–6]. Underrepresentation of AA in PD research and clinical care likely contributes to these health disparities [7–10]. To increase health equity and reduce health disparities among people with PD, we need to improve the representation of AA in PD care and research. Several factors may contribute to the underrepresentation of AA in current PD research and care, including AA’s historical mistrust of healthcare systems, providers, and researchers; lack of insurance; insufficient education about research opportunities and the research process; and provider bias [11,12]. These factors suggest that the underrepresentation of AA with PD originates from complex, multi-level issues. While the existing literature provides general insights into this issue, it does not directly inform how to address underrepresentation and improve engagement in research, clinical care, and community services at the local level. Understanding the perspectives of AA with PD, their care partners, and their providers from local communities may reveal specific priorities and needs, inform effective practices to diversify PD research, and ultimately enhance health equity among AA with PD [9,13–15].

Community-engaged research, which involves collaborating with community members throughout the research process, is one such way to do so [16]. In particular, the Community Engagement (CE) Studios method is a promising qualitative, consultative method to collaborate with community members to incorporate their expertise into enhancing research design, execution, and dissemination [17,18]. CE Studios position community members as experts in their lived experiences and seeks their consultation to develop and enhance research processes and establish future directions [17,18]. Although they can be used at any stage of research, it is encouraged that CE Studios be conducted in early stages of research idea development to make research processes more equitable and culturally appropriate [17,18]. This method has proven feasible and beneficial in other clinical populations like Alzheimer’s disease, but it has not yet been used in PD research [19–21].

In this paper, we showcase the use of CE Studios as a community-engaged research method to inform the design and execution of a planned research project aiming to understand engagement in PD care, research, and community services among AA individuals. Specifically, we used CE Studios to (1) guide the development of our upcoming interview study by uncovering priority barriers and facilitators to engagement, (2) identify key stakeholders and partners to support study recruitment, and (3) refine study materials and methods to optimize appeal and cultural appropriateness. Findings from these CE Studios can contribute to improving the cultural appropriateness of the proposed upcoming research, ultimately helping to produce important evidence on improving health equity in PD. Furthermore, our work can provide a practical example of using CE Studios to promote a diverse, equitable, and inclusive PD research process in other local contexts.

## Methods

### Overview

We conducted three CE Studios at Washington University in St. Louis (WashU). We held one studio with AA individuals with PD and their care partners, one with healthcare providers for AA individuals with PD, and one with all three groups (people with PD, care partners, healthcare providers). We provide more details about each studio below. By utilizing multiple note-takers and coders, we sought to ensure a comprehensive and accurate execution and analysis of the project. While this type of in-depth analysis is not required in CE Studios, we used this approach to gain a deeper understanding of the perspectives of community partners. Because this was a quality improvement project, we did not need IRB approval or informed consent.

### Community engagement (CE) studios participant eligibility and recruitment

For AA individuals with PD, we recruited those who were 18 years old or older and had PD. For care partners, we recruited individuals who were 18 years old or older and were past or current care partners for AA with PD. For healthcare providers, we recruited those who had previously or were currently providing care for with AA individuals with PD. Participants were recruited through word of mouth and referrals.

### Community engagement (CE) studios

We conducted three CE Studios: Studio 1 was in-person and included AA people with PD (*N* = 4) and care partners (*N* = 2); Studio 2 was virtual (Zoom) and included healthcare providers of AA with PD (*N* = 8; 3 primary care physicians, 3 neurologists, 1 occupational therapist, 1 physical therapist, 1 nurse); and Studio 3 was virtual and included a mix of all three groups (*N* = 4; 1 AA with PD, 1 care partner, 1 counselor, 1 nurse). CE Studios were facilitated by a neutral expert in qualitative, community-engaged research who identifies as AA (JBB) and included the following components: 1) introductions, 2) brief presentation by the research team about the research goals, and 3) facilitated discussion around key topic areas. All studios were confidential and were not video or audio recorded. Three to six trained researchers served as note takers at every studio to capture the qualitative data for analysis. We developed the semi-structured interview guide using the perceived barriers and perceived benefits constructs from the Health Belief Model [22].

The overarching purpose of all CE Studios was to inform the design and process of a future interview research study in consultation with relevant community members. The goal of the first two studios was to gain insights into their experiences and needs associated with engaging in PD-related research, clinical care, and community services. This was for the purpose of developing an interview guide that addresses common and high-priority areas. Specific topics discussed included barriers and facilitators to engagement in clinical care, research, and community services, as well as stakeholders and partners to engage in this work. Example prompts used during the studios include, “What are culturally appropriate ways for our group to engage with Black and African American communities?” and “We want to increase engagement among Black and African American people with PD. What helps with or promotes engagement with research, clinical care, and community services?” The goal of the third studio was to seek feedback from community members to ensure the materials and methods for the upcoming interview study are culturally appropriate. The specific topics discussed included the procedures (e.g., recruitment methods, remuneration, interview conduct) of the planned qualitative interview study.

### *National Institute on Aging Health Disparities Research Framework* ^23^

We used the National Institute on Aging Health Disparities Research Framework to inform the analysis of the findings from the three CE Studios. This framework describes the key factors related to health disparity across levels including environmental, sociocultural, behavioral, and biological. Table 1 describes the details of the framework.

**Table 1.** National institute on aging health disparities research framework

Levels	Factors		
Environmental	Geographical and political factors (e.g., structural bias, residential segregation)	Socioeconomic factors (e.g., education, income)	Health care (e.g., access, literacy)
Sociocultural	Cultural factors (e.g., prejudice, norms)	Social factors (e.g., social network, institutional racism)	Psychological factors (e.g., self-concepts, stigma)
Behavioral	Coping factors (e.g., active coping, emotional regulation)	Psychosocial risk/resilience (e.g., social support, discrimination)	And health behaviors (e.g., drug, nutrition)
Biological	Physiological indicators (e.g., co-morbidities, inflammation)	Genetic stability (e.g., loss of proteostasis, epigenetic alteration)	Cellular function and communication (e.g., cellular senescence, stem cell exhaustion)

### Analysis

We used both inductive and deductive content analysis to analyze the notes taken during the three studios and create themes and codes [24–26]. First, three authors (EK, DW, JY) independently coded Studios data as 1) barriers and facilitators, 2) community partners to engage with, and 3) suggestions to improve the upcoming interview study research using deductive content analysis and then further coded them using inductive approach [22] From the first studio, we generated three sets of notes. Coders (EK, DW, JY) each independently analyzed one set of notes and then developed a preliminary codebook. Following this, the coders conducted consensus coding to refine and synthesize the preliminary codebook into a final version. When there was a discrepancy, we consulted with the team and senior authors (JBB and EF). For the second and third studios, we generated three and six sets of notes, respectively. The second studio was analyzed by EK, DW, and KD; the last one was analyzed by EK, JY, and DW. We used the same analysis procedures across all three studios. After coding each studio’s data, we combined all codes and further analyzed them using deductive content analysis based on the National Institute on Aging Health Disparities Research Framework [23]. One coder (EK) independently coded all data and then conducted consensus coding with the team to finalize the results. When there was a discrepancy, we consulted with the team and senior authors (JBB and EF).

To improve the trustworthiness of our analysis, we completed the following tasks.27 By having multiple coders and senior authors, we improved the credibility of our analysis. We specified the backgrounds of the researchers above in consideration of the reflexivity of this work. As we had multiple coders and used the National Institute on Aging Health Disparities Research Framework as a guide, we do not believe that our experience as individuals had substantial impacts on analyzing the data. Having a detailed description of this project process, analysis methods, and findings including example quotes helped us improve transferability, dependability, and confirmability of this work. While the analysis used in this project is not a requirement for all CE Studios, this approach suggests an alternative way to understand the perspectives of community partners.

## Results

### Participant demographics

Table 2 describes the demographics of the participants. Among individuals with PD and care partners, all were Black or African American, with both female and male participants. In the healthcare provider group, we had diverse participants in terms of sex and race.

**Table 2.** Participant characteristics

	People with PD and Care partners (*N* = 8)	Healthcare providers (*N* = 10)
Age (median)	70.5	Not available
Sex (Female/Male)	5/3	5/5
Race		
Asian	0	3
Black or African American	8	5
White	0	2
Ethnicity (Not Hispanic or Latin/Hispanic or Latin)	(6/0*)	(10/0)

*One person declined to state their ethnicity, and one person did not answer this survey item.

### Overview

We identified 1) barriers and facilitators to engagement in PD research, clinical care, and community services and suggestions for future works and 2) refined the upcoming study materials and methods.

### Barriers to engagement

Participants identified barriers to engagement in PD research, clinical care, and community services in all levels of the NIA Health Disparities Research Framework (environmental, sociocultural, behavioral, and biological) (Table 3).

**Table 3.** Health disparity factors influencing AA participation in PD research, clinical care and community services

	Barriers	Facilitators
Environmental		
Healthcare	Limited accessibility to research, clinical care, and community services Lack of knowledge among African Americans (AA) with Parkinson’s Disease (PD) Providers’ lack of knowledge and awareness Financial limitations Poor healthcare quality Medical mistrust Insufficient or inadequate healthcare resources	Adequate knowledge among AA with PD Providers’ knowledge and awareness Availability of financial resources High-quality healthcare Medical trust Adequate healthcare resources
Geographical and political	Accessibility Dehumanization Historical mistrust Systematic segregation	–
Socioeconomic	Lack of or inadequate financial resources/support	Adequate financial resources/support
Biological		
Physiological indicators	Symptoms	–
Sociocultural		
Cultural	PD stigma in AA community Lack of communication in AA community	AA and their community empowerment Develop AA community role model Respecting AA community
Psychological	Emotional barriers to access academic medical center Fear being perceived as a disabled person	–
Social	A lack of proper support from AA community Not being invited Poor representation of AA community in PD	Care partner support
Behavioral		
Coping	Lack of communication in AA community Denial	AA and their community empowerment
Psychosocial risk/resilience	Lack of social support	Adequate social support AA and their community empowerment

#### Environmental level

*Health care factors.* Multiple barriers were related to healthcare access including limited accessibility of research studies and community organizations and the absence of referrals to specialized care from local providers. Quality of health care was also mentioned, emphasizing limited PD-specific knowledge and awareness among both AA and providers and a lack of culturally appropriate/tailored education/information for AA with PD. Other identified barriers included financial transparency related to clinical care and research and inadequate insurance.

“WashU is viewed as a top tier institution, seen as almost gated from St Louis, inaccessible” – *AA with PD or care partner from Studio 1*

*Geographical and political factors.* Several geographical access-related barriers were identified, including general accessibility and transportation. Additionally, issues of systematic segregation, historical mistrust, and dehumanization were recognized.

“Historical context, don’t want to be seen as guinea pigs, push pins.” – *AA with PD or care partner from Studio 1*

“Segregation has caused a huge barrier to connect with the AA community.” – *AA with PD or care partner from Studio 1*

*Socioeconomic factors.* A lack of financial resources among the AA community was suggested as a socioeconomic barrier.

“Many times, money is a limiting factor” – *AA with PD or care partner from Studio 1*

#### Sociocultural level

*Cultural factors.* Cultural barriers included PD stigma and hesitancy to communicate about PD within the AA community.

“We as Blacks will say ‘I’m just getting older.’ PD can be perceived as normal aging.”– *AA with PD or care partner from Studio 1*

“We do not like to talk about it! In church, when you disclose you have PD, people respond, ‘I do not need to know about that.’”– *AA with PD or care partner from Studio 1*

“We should ask especially the young people if they feel a stigma attached to the disease, because especially young people tend to hide the disease from their friends & family.” – *Provider from Studio 2*

*Social factors.* Social barriers included the AA community not being invited, underrepresentation in PD, and a lack of referrals to WashU from local providers.

“People weren’t necessarily invited to the table to begin with. Communication hasn’t been directed to Black community, so they don’t feel a part of it. Black people living with PD may feel like an anomaly.” - *Provider from Studio 2*

*Psychological factors.* Emotional barriers preventing access to WashU among the AA community and concerns over being perceived as disabled were identified.

“WashU is known as a premier educational institution that is almost gated from St Louis. It needs to be viewed as more approachable rather than gated.” – *AA with PD or care partner from Studio 1*

“My feeling about that is that sometimes that takes us back to previous research studies and things that are still not comfortable for our communities discussing. So what message is it sending that (our institution)’s name is at the top of the flyer.” – *AA with PD, care partners, or providers from Studio 3*

#### Behavioral level

*Coping.* Identified barriers included the AA community’s cultural hesitancy to communicate about PD and denial about having PD.

“AA community needs to be more transparent about their symptoms and their conditions.” – *AA with PD or care partner from Studio 1*

*Psychosocial risk/resilience.* A lack of social support for and from the AA community were highlighted.

“Less social support for, e.g., transportation to come to research studies” - *Provider from Studio 2*

*Physiological indicators.* PD symptoms were noted as factors hindering those with PD from engaging in research, clinical care, and community services.

“Depends on symptom severity, cognitive function.” - *Provider from Studio 2*

### Facilitators

Facilitators covered environmental, sociocultural, and behavioral levels but not the biological level (Table 3).

#### Environmental level

*Health care.* Multiple health education- and literacy-related facilitators were identified. These included the need for PD-specific content, insurance, financial resources, and care partner-related information. The importance of clear, diverse, culturally appropriate, and tailored educational content for the AA community was suggested.

“Need a movement to make (PD) diagnosis well known, like stroke, especially to people of minority backgrounds – like a public health campaign and make sure info is getting relayed specifically to minority population” - *Provider from Studio 2*

The variety of channels for education dissemination was mentioned, including doctors, insurance, government agencies, civic organizations, and nurses, available both virtually and in-person. Other codes related to healthcare accessibility included access to healthcare, WashU resources, referrals to WashU for PD care, and referrals to PD organizations. Quality of healthcare-related codes highlighted providing sufficient clinical time for AA, research transparency, safety, community collaboration, engaging the AA community throughout all research phases, mutually beneficial research, and promoting equal partnership in the research process.

“Getting people involved in not just being research participants but in building the research process; helping to determine which direction the research goes; so that people don’t feel like things are being done ‘to them’” – *Provider from Studio 2*

Additional identified codes emphasized insurance, medication assistance, WashU’s role in educating local providers, community services, leveraging existing partnerships, and making WashU approachable to the AA community.

“Wash U as an educational institution needs to be more approachable, provide classes online to educate the community, provide educational opportunities that are helpful to the black community in a fun way and teach in more diverse ways.” – *AA with PD or care partner from Studio 1*

*Socioeconomic factors.* Financial resources and support were highlighted as facilitators.

“Programs to offset the cost of transportation” - *Provider from Studio 2*

#### Sociocultural level

*Cultural and Social factors.* The empowerment of AA with PD and their community emerged as both cultural and social facilitators. Within cultural factors, promoting AA community role models and respecting the AA community were also emphasized. Within social factors, the importance of care partner support was highlighted.

“Let the community tell you what is needed” - *Provider from Studio 2*

“Helping people & care partners feel like they’re not alone in their disease process or in their struggles; helping them feel a part of something” - *Provider from Studio 2*

#### Behavioral level

*Coping Factors and Psychosocial Risk/Resilience.* Empowerment of the AA community was recognized as a facilitator in both the coping and psychosocial risk/resilience categories.

“Leaving a legacy behind…knowledge will benefit generations in the future (children) – although this is a hard sell for traditionally underrepresented groups because they feel like they haven’t benefitted from past research” - *Provider from Studio 2*

### Community partners to engagement

Participants recommended collaborating with personnel from adult day care, civic organizations, medical practices, faith/spiritual entities, high schools, insurance firms, local government agencies, nursing, and provider sectors. Environments for potential engagement included adult care centers, civic groups, community health services, faith/spiritual establishments, government bodies, health clinics, homecare services, local Federally Qualified Health Centers, local governmental agencies, long-term care facilities, and senior living communities.

### Feedback on upcoming PD interview study designs and methods

**Figure 1.**
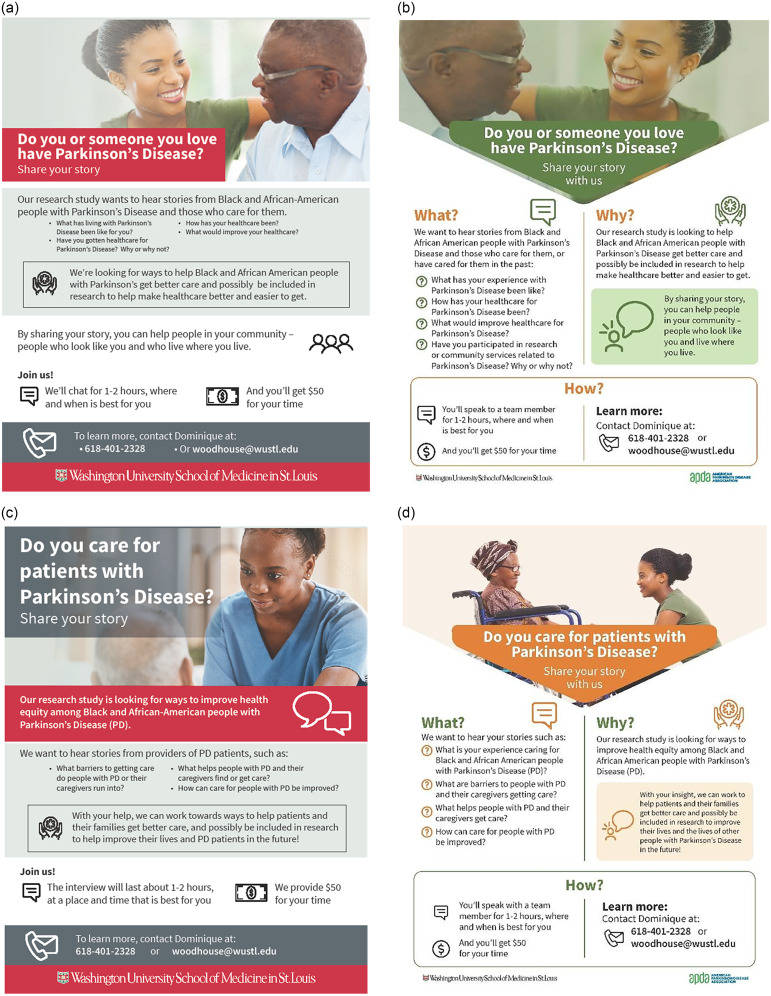
Initial and refined recruitment materials. a. Initial patient and care partner recruitment flyer draft, b. Refined final patient and care partner recruitment flyer after CE studios, c. Initial provider recruitment flyer draft, d. Refined final provider recruitment flyer after CE studios.

Participants suggested feedback regarding our upcoming PD research study designs, especially pertaining to interview methods, duration, and recording. Specifically, they recommended offering options to opt in or out of interview recordings, ensuring research confidentiality, conducting separate interviews for AA with PD and their care partners, emphasizing training for interviewers, conducting interviews in the format and location of the participant’s preference (e.g., in-person at their home or the university, phone, Zoom), and limiting interviews to 1-2 hours. Additionally, they provided input into the design of our recruitment materials (Figure 1). The participant feedback ranged from design features, such as using a green color theme to make it more eye-catching, to making the flyer simple, personal, and inviting. Additionally, the feedback included incorporating inclusive language for previous and current care partners (e.g., “Do you/have you care(d) for a person with Parkinson’s Disease?”) and clarifying research study activities (e.g., clarifying the availability of a virtual interview option).

## Discussion

In this project, we used CE Studios to improve the representation of the AA community in a planned PD qualitative interview study, as well as to inform our future research. We identified priority barriers and facilitators to engagement in our local context, encompassing environmental, sociocultural, behavioral, and biological dimensions, and we received concrete input on specific methodological aspects of our planned study.

The CE Studios highlighted both general and PD-specific health disparity factors, such as transportation and access to research and care, historical mistrust, segregation, and the importance of culturally appropriate education in PD. Such factors are recognized as pivotal for engagement in both research and care, not just among AA with PD but also in other populations [11,12,19].

Our results also highlighted PD-specific barriers and facilitators consistent with existing evidence [11,12,28]. For example, participants indicated that PD symptoms such as cognitive impairment and slowness of movement can prevent them from engaging in clinical care, research, and community services. Moreover, various factors contribute to the reluctance, inability, or unwillingness of AA with PD to participate in these areas. These factors include a lack of awareness and understanding of PD within the AA community, insufficient representation of the AA community in PD, the lack of culturally relevant and tailored education, biases or knowledge gaps among healthcare providers about AA with PD, and problems such as missed, incorrect, or delayed PD diagnoses in AA.

The CE Studios also uncovered factors relevant to our local region. For example, participants highlighted issues such as residential segregation, difficulties in accessing local clinical care, research institutions, community venues, and inadequate transportation. Most facilities that provide specialized PD care, research, or community assistance are located far from where the majority of AA reside. Moreover, local providers seldom refer AA patients with PD to our academic medical institution, which offers a variety of specialized care, research, and connection with community services. As a result, AA patients with PD are often unaware of or unable to access the specialized PD care, research opportunities, and community resources available to them. Beyond these environmental challenges, there are also sociocultural and behavioral barriers. Some individuals feel hesitant about approaching our institution due to negative past experiences or mistrust. It was also noted that local researchers often do not put forth much effort to engage with AA communities perceived as “hard to reach.” Participants voiced that our institution should be proactive in educating local providers, fostering better community collaboration, and delivering PD education specifically tailored for the AA community.

The CE Studios informed the refinement of our study designs based on these regional insights. For example, we refined the semi-structured interview guides (e.g., shortened, tailored questions to each participant group), recruitment materials, and methods (e.g., flexibility in scheduling and mode of interviews). This locally specific knowledge enhanced our understanding of the community and informed the adaptation of our research process to better meet their specific needs. This aligns with previous findings on the utility of CE Studios in promoting equitable research processes and diversifying research participation, specifically within the priority region or population [19–21].

Informed by the perspectives and feedback from the participants, we suggest action steps to overcome barriers and leverage facilitators. Some barriers, such as transportation and a lack of culturally appropriate research processes or recruitment materials, can be addressed with relative ease through individual or organizational-level efforts. For instance, solutions might encompass budgeting for participant transportation and tailoring study materials. Novel research recruitment methods and PD education using non-traditional, more accessible approaches should also be explored, given their potential in reaching and recruiting diverse and broader populations [29–32]. Conversely, barriers like the poor referral rate for research participation in the AA community, PD stigma in the AA community, historical mistrust, systematic segregation, and others demand not only individual and organizational efforts but also broader, systematic, and multi-level interventions. One of these barriers, the providers’ poor referral rate for research among the AA community, has been tackled in the past through the intervention targeting providers [33]. However, this intervention was not effective, underscoring the absence of evidence-based approaches [33]. While we advocate for the development of comprehensive, multi-level strategies to address these issues, we also recommend persisting with actionable solutions at the individual and organizational levels.

This project also offers important implications for optimizing the process of CE Studios in future PD and other research. We observed varying levels of participant engagement in the studios, with some individuals being more vocal than others. Consequently, the facilitator had to make a deliberate effort to engage all participants, ensuring every voice was heard. Moreover, customizing CE Studios can bolster diverse participation. In our project, for instance, providers generally favored virtual participation, whereas AA individuals with PD and their care partners preferred in-person interactions. Providing necessary and tailored accommodations (e.g., CE Studios for Spanish speakers or providing family/childcare services during sessions) may further enhance the diversity of CE Studios participation and promote equitable research practice.

### Limitations

Due to the small sample, it is unlikely that our CE Studios participants fully represent the diverse AA PD community in the region. Importantly, within the AA community, perspectives can vary widely since different sub-groups might have distinct values and experiences. This project primarily focused on gathering input from community partners as part of the CE Studios and did not engage them in the earlier or later phases of the process, such as developing research questions, analyzing and synthesizing the findings, member checking, and disseminating the results through peer-reviewed publications, due to logistical limitations. In future work, we aim for a more active and engaging form of community involvement, from the inception of the process to the final dissemination of the findings.

## Conclusion

This project supports that the CE Studios method is a valuable community-engaged research method and extends its application to the PD context. By demonstrating its usefulness in providing insights into community partners’ perspectives on the research process, we enhance the cultural relevance and responsiveness of research studies to the needs of the PD community. Our findings make a unique contribution to PD research by validating the use of CE Studios in this population-specific context. In addition, we highlight PD-related health disparities across diverse levels, underlining the need for comprehensive, multi-level strategies to achieve health equity. We encourage future studies to actively use community-engaged methods like CE Studios to identify priority health disparity factors in their local contexts to inform more equitable and inclusive research practices.
